# Genomic Prediction and Association Analysis with Models Including Dominance Effects for Important Traits in Chinese Simmental Beef Cattle

**DOI:** 10.3390/ani9121055

**Published:** 2019-12-01

**Authors:** Ying Liu, Lei Xu, Zezhao Wang, Ling Xu, Yan Chen, Lupei Zhang, Lingyang Xu, Xue Gao, Huijiang Gao, Bo Zhu, Junya Li

**Affiliations:** 1 Laboratory of Molecular Biology and Bovine Breeding, Institute of Animal Sciences, Chinese Academy of Agricultural Sciences, Beijing 100193, China; yliu2333@sina.com (Y.L.); xuleirock@163.com (L.X.); wangzezhao1@163.com (Z.W.); jiujiuyake@sina.com (L.X.); chenyan0204@163.com (Y.C.); zhanglupei@caas.cn (L.Z.); xulingyang@163.com (L.X.); gaoxue76@126.com (X.G.); gaohj111@sina.com (H.G.); 2 Institute of Animal Husbandry and Veterinary Research, Anhui Academy of Agricultural Sciences, Hefei 230031, China; 3 Animal Science and Technology College, China Agricultural University, Beijing 100193, China; 4 National Centre of Beef Cattle Genetic Evaluation, Beijing 100193, China

**Keywords:** dominance, variance components, genomic prediction, GWAS, Simmental beef cattle

## Abstract

**Simple Summary:**

Dominance effects play important roles in determining genetic changes with regard to complex traits. We conducted genomic predictions and genome-wide association studies in order to investigate the effects of dominance on carcass weight, dressing percentage, meat percentage, average daily gain, and chuck roll in 1233 Simmental beef cattle. Using dominance models, we improved the predictive abilities and found several candidate single-nucleotide polymorphisms (SNPs) and genes associated with these traits. Our studies helped us to understand causal mutation mapping and genomic selection models with dominance effects in Chinese Simmental beef cattle.

**Abstract:**

Non-additive effects play important roles in determining genetic changes with regard to complex traits; however, such effects are usually ignored in genetic evaluation and quantitative trait locus (QTL) mapping analysis. In this study, a two-component genome-based restricted maximum likelihood (GREML) was applied to obtain the additive genetic variance and dominance variance for carcass weight (CW), dressing percentage (DP), meat percentage (MP), average daily gain (ADG), and chuck roll (CR) in 1233 Simmental beef cattle. We estimated predictive abilities using additive models (genomic best linear unbiased prediction (GBLUP) and BayesA) and dominance models (GBLUP-D and BayesAD). Moreover, genome-wide association studies (GWAS) considering both additive and dominance effects were performed using a multi-locus mixed-model (MLMM) approach. We found that the estimated dominance variances accounted for 15.8%, 16.1%, 5.1%, 4.2%, and 9.7% of the total phenotypic variance for CW, DP, MP, ADG, and CR, respectively. Compared with BayesA and GBLUP, we observed 0.5–1.1% increases in predictive abilities of BayesAD and 0.5–0.9% increases in predictive abilities of GBLUP-D, respectively. Notably, we identified a dominance association signal for carcass weight within *RIMS2*, a candidate gene that has been associated with carcass weight in beef cattle. Our results suggest that dominance effects yield variable degrees of contribution to the total genetic variance of the studied traits in Simmental beef cattle. BayesAD and GBLUP-D are convenient models for the improvement of genomic prediction, and the detection of QTLs using a dominance model shows promise for use in GWAS in cattle.

## 1. Introduction

Dominance is a result of interactions between genes at the same locus, and it plays an important role in mammalian biology and development [1,2]. Several studies have been conducted to decompose dominance genetic effects from the total genetic variance of complex traits. For instance, Vitezica et al. [3] estimated the variance components including additive and dominance variance in 1184 mice, and the dominance variance accounted for 10.5–43.3% of the genetic variance for the studied traits. In addition, dominance variance was found to account for 5% and 7% of the total variance for milk yield traits in the US Holstein and Jersey populations, respectively [4]. For nine different beef cattle populations, the average proportion of dominance variance to phenotypic variance was equal to 5% across 16 growth, carcass, and fertility traits [5]. 

Dominance effects are important non-additive genetic effects and the inclusion of the dominance effects in prediction models could increase the accuracy of genomic prediction in farm animals. Nishio et al. [6] conducted genomic prediction using genomic best linear unbiased prediction (GBLUP) including dominance effects in pigs, and found that the accuracy of estimated breeding values for GBLUP-D outperformed GBLUP by over 1.2% for average daily gain. Some researchers have presented several datasets for mapping QTLs with non-additive effects. For example, several SNPs located on BTA14 were found to have the largest dominance effects for fat yield in US Holsteins and Jerseys populations [4]. Jiang et al. [7] identified and validated a dominance association signal with milk yield near *RUNX2*, a candidate gene that has been associated with milk production in dairy cattle. In pigs, SNPs located in SSC7 with significant dominance effects for the number of teats were also reported [8]. However, in Simmental beef cattle, we are not aware of studies on genomic prediction and genome-wide association including dominance effects.

Simmental is one of the important introduced beef cattle breeds in China. In this study, we analyzed five economically important traits (carcass weight, dressing percentage, meat percentage, average daily gain, and chuck roll) in 1233 Simmental beef cattle genotyped with BovineHD Beadchip. Some traits are important in beef cattle production systems and considered as main objectives of the genetic improvement. For example, average daily gain contributes to production efficiency and economic benefits in the beef cattle industry [9]. Carcass weight is an important indicator for growth status, and dressing percentage and meat percentage generally imply a large proportion of the live weight [10]. The aims of this study were to (1) estimate the relative contribution of additive and dominance effects for these traits, (2) identify significant dominance signals, and (3) investigate whether models including dominance effects can improve the predictive ability for economically important traits.

## 2. Materials and Methods

### 2.1. Ethics Statement

All animals used in the study were treated following the guidelines established by the Council of China Animal Welfare. Protocols of the experiments were approved by the Science Research Department of the Institute of Animal Sciences, Chinese Academy of Agricultural Sciences (CAAS) (Beijing, China). The approval ID/permit numbers are SYXK (Beijing) 2008-007 and SYXK (Beijing) 2008-008.

### 2.2. Animals and Phenotypes

A total of 1302 Simmental beef cattle born between 2008 and 2013 were used in this study, and these cattle originated from Ulgai, Xilingol League and Inner Mongolia, China. After weaning, the cattle were moved to JinweifurenCo, Ltd., Beijing, China for fattening. All animals shared the same feeding and management conditions. A more detailed description of the breeding and management has been described previously [11,12]. The cattle were slaughtered at an average age of 20 months. During the period of slaughtering, we measured traits in strict accordance with the guidelines proposed by the Institutional Meat Purchase Specifications for fresh beef [13]. In this study, we analyzed five traits, namely, carcass weight (CW), dressing percentage (DP), meat percentage (MP), average daily gain (ADG), and chuck roll (CR). Summary statistics of the five traits are listed in Table 1.

### 2.3. Genotyping and Quality Control

We extracted genomic DNA from blood samples using the TIANamp Blood DNA Kit (Tiangen Biotech Co. Ltd., Beijing, China). DNA quality was considered acceptable if the A260/280 ratio (the ratio of absorbance at 260 nm and 280 nm made on a spectrophotometer) was 1.8–2.0. Qualified DNA was genotyped using the Illumina BovineHD Beadchip panel (Illumina Inc., San Diego, CA, USA). The SNP chips were analyzed with Infinium GenomeStudio (Illumina). Before statistical analysis, SNPs were pre-processed using PLINK v1.90 [14]. SNPs were selected based on minor allele frequency (>0.05), proportion of missing genotypes (<0.05), and Hardy–Weinberg equilibrium (*p* > 10^−6^). Moreover, individuals with more than 10% missing genotypes were excluded. After these quality controls, the final data consisted of 1233 individuals and 596,978 (out of 180,984) autosomal SNPs.

### 2.4. GBLUP-D

The linear mixed-model including additive and dominance genetic effects can be written as follows:

(1) $$y=Xb+Z_{a}a+Z_{d}d+e,$$

where **y** is the vector of phenotypes; **b** is the vector of fixed effects including year, sex, and body weight at entering the fattening farm, and the number of fattening days. Random additive genetic effects **a** and random dominance effects **d** are related to **y** by incidence matrices $Z_{a}$ and $Z_{d}$ respectively, and **e** is the vector of random residuals. 

It is assumed that

(2) $$a\sim N\left(0,G\sigma_{a}^{2}\right);\;d\sim \left(0,D\sigma_{d}^{2}\right);\;e\sim N\left(0,I\sigma_{e}^{2}\right),$$

where$\;\sigma_{a}^{2}\;$is the additive genetic variance, $\sigma_{d}^{2}$ is the dominance variance, $\sigma_{e}^{2}$ is the residual variance, **I** is an identity matrix, and **G** and **D** are the additive and dominance genetic relationship matrices, respectively. In this study, these matrices were constructed using SNP information as follows:

The additive genomic relationship matrix G was constructed using SNP marker information according to the previous study [1]. In short, $G=\frac{MM\prime }{\sum 2p_{i}q_{i}}$, where **M** is an n × m matrix (n = number of animals and m = number of marker loci), which refers to SNP genotype coefficients at each locus. The coefficients of the ith column in the **M** matrix are ($0\;-\;2p_{i}$) for genotype $A_{1}A_{1}$, ($1-2p_{i}$) for $A_{1}A_{2}$, and ($2-2p_{i}$) for $A_{2}A_{2}$, where $p_{i}$ and $q_{i}$ are the frequencies of allele 1 ($A_{1}$) and allele 2 ($A_{2}$) at locus i, respectively.

The dominance genomic relationship matrix is $D=\frac{HH\prime }{\sum 2p_{i}q_{i}\left(1-2p_{i}q_{i}\right)}$ [1]. For n individuals and m loci, **H** is the n × m matrix of heterozygosity coefficients with element $h_{\mathrm{ki}}=0-2p_{i}q_{i}$ if individual k is homozygous, and $h_{\mathrm{ki}}=1-2p_{i}q_{i}$ if individual k is heterozygous at locus i. By centering $h_{\mathrm{ki}}$ with $2p_{i}q_{i}$ and scaling **HH**’ with $\sum 2p_{i}q_{i}\left(1-2p_{i}q_{i}\right)$, the **D** matrix has the properties that the expectation of an off-diagonal element is zero for two unrelated individuals, and the expectation of a diagonal element is one for a non-inbred individual [1]. In this study, genomic breeding values and additive and dominance variance components were computed using the ASReml package [15].

### 2.5. BayesAD 

The linear mixed-model including additive and dominance SNP effects can be written as follows:

(3) $$y=Xb+W_{a}\alpha +W_{\beta }\beta +e,$$

where **y** is the vector of phenotypes observations; **b** is the vector of fixed effects including year, sex, and body weight at entering the fattening farm and the number of fattening days; α and β are the vectors of SNP additive and dominance effects, respectively; $W_{\alpha }$ is incidence matrix of SNP additive effects that take the values 0, 1, and 2 for the SNP genotypes AA, Aa, and aa at each loci, respectively; and $W_{\beta }$ is incidence matrix of SNP dominance effects that take the values 0, 1, and 0 for the SNP genotypes AA, Aa, and aa, respectively. **e** is the vector of random residuals. 

The BayesAD assumes that the marker additive effects follow Student’s *t*-distribution, that is, the normal distribution of variance following the scaled inverse chi-square distribution [16]. The absolute value of the additive effects $\left|\alpha_{j}\right|$ following a folded *t*-distribution has a half-normal distribution, since it is conditional on an inverse chi-square distributed parameter $\sigma_{\alpha_{j}}^{2}$. b and $\sigma_{e}^{2}$ both follow uniform distribution. The model can also be described as follows [16]:

(4) $$\left|\alpha_{j}|\left|\sigma_{\alpha_{j}}^{2}\sim \left(iid\right)\right|N\left(0,\sigma_{\alpha_{j}}^{2}\right)\right|\;j\;=\;1,\;\ldots ,\;m$$

(5) $$\sigma_{\alpha_{j}}^{2}|\chi^{-2}\left(v_{\alpha },s_{\alpha }^{2}\right)$$

(6) $$\beta_{j}|\left|\alpha_{j}\right|,\sigma_{\beta_{j}}^{2}\sim N\left(\mu_{\beta }\left(\left|\alpha_{j}\right|\right),\sigma_{\beta }^{2}\left(\left|\alpha \right|,\sigma_{\alpha_{j}}^{2}\right)\right)$$

The traditional BayesA only has additive effects in the model. Therefore, $\mu_{\alpha }\left(\left|\alpha_{j}\right|\right)=0$ and $\mathrm{var}\left(\alpha_{j}\right)=E\left(\sigma_{\alpha_{j}}^{2}\right)=s_{\alpha }^{2}\frac{v_{\alpha }}{v_{\alpha }-2}$ where $v_{\alpha }$ is the degree of freedom and $s_{\alpha }^{2}$ is the scale parameter in the scaled inverse chi-square distribution for the variance of marker effects. Assuming that the additive effects and the dominance effects are independent in BayesAD, that is, $\mu_{\beta }\left(\left|\alpha_{j}\right|\right)=\mu_{\beta },\;\;\sigma_{\beta }^{2}\left(\left|\alpha \right|,\sigma_{\alpha_{j}}^{2}\right)=s_{D}^{2}\sigma_{\alpha_{j}}^{2}$ where $s_{D}>0$. The additive SNP effects estimation is the same as in BayesA.

The Markov chain for the prediction of the model parameters is generated by Gibbs sampling with a Metropolis–Hastings (MH) step [16]. According to Bayesian theory, the complete condition posterior distribution of each variable is derived as follows [16]:

When given y, the joint posterior distribution of b,  α, $\beta ,\;\sigma_{\alpha }^{2},\;and\;\sigma_{e}^{2}$ is

(7) $$f\left(b,\theta ,\sigma_{\alpha }^{2},\sigma_{e}^{2}|y\right)\propto f\left(b,\theta ,\sigma_{\alpha }^{2},\sigma_{e}^{2}\right)f\left(y|b,\theta ,\sigma_{\alpha }^{2},\sigma_{e}^{2}\right)$$

(8) $$\propto f\left(y|b,\theta ,\sigma_{\alpha }^{2},\alpha_{e}^{2}\right)f\left(b\right)f\left(\theta |\sigma_{\alpha }^{2}\right)f\left(\sigma_{\alpha }^{2}\right)f\left(\sigma_{e}^{2}\right)$$

f(b), $f\left(\theta |\sigma_{\alpha }^{2}\right),$$f\left(\sigma_{\alpha }^{2}\right),$ and $f\left(\sigma_{e}^{2}\right)$ are the prior distributions of b, θ=(α,β), $\sigma_{\alpha }^{2},$ and $\sigma_{e}^{2}$, respectively. The priori of the SNP dominance effect $\beta_{j}$ is a conditional prior to the absolute value $\left|\alpha_{j}\right|$. Therefore, it is necessary to construct a joint posterior distribution of $\beta_{j}$ and $\left|\alpha_{j}\right|$ for obtaining the sample value by MH sampling [16]. Given the joint prior distribution of $\beta_{j}$ and $\left|\alpha_{j}\right|$:

(9) $$f\left(\theta_{j}|\sigma_{\alpha_{j}}^{2}\right)=f\left(\left|\alpha_{j}\right||\sigma_{\alpha_{j}}^{2}\right)f\left(\beta_{j}|\left|\alpha_{j}\right|,\sigma_{\alpha_{j}}^{2}\right)$$

(10) $$f\left(\beta_{j}|\alpha_{j},\sigma_{\alpha_{j}}^{2}\right)=\frac{1}{\sqrt{2\pi \sigma_{\beta_{j}}^{2}}}exp\left(-\frac{{\left(\beta_{j}-\mu_{\beta }\right)}^{2}}{2\sigma_{\beta_{j}}^{2}}\right)$$

### 2.6. Genome-Wide Association Studies based on Dominance Effects

The association mapping of dominance effects and additive effects was performed using a multi- locus mixed-model (MLMM) approach in R [17]. This approach involves a stepwise mixed-model regression with forward inclusion and backward elimination of genotypic markers included as fixed effects. This model can potentially improve the power, whilst also reducing the rate of false positives in genome-wide association studies (GWAS) [18]. Moreover, for the significant SNPs, the UMD3.1 genome assembly was used to locate genes for annotation [19]. Candidate genes containing significant SNPs were listed. If the SNPs were not located within genes, the closest gene (either on the 5′ or 3′ end) was identified with 100 kb between the SNP and the gene.

### 2.7. Model Validation and Predictive Ability

Goodness of fit for GBLUP-D and GBLUP was assessed by the likelihood value. The superiority of GBLUP-D over GBLUP was also tested using a likelihood ratio test. Moreover, a five-fold cross validation was used to evaluate the accuracy of genomic prediction for additive genetic values in both GBLUP-D and BayesAD. Data were split into five approximately equal-sized groups. For each cross validation, four groups were used as the training sample to estimate the marker effects, and the remaining group was used as the validation sample. The cross-validation procedure was repeated 25 times. Predictive ability was assessed by the correlation between predicted genetic values and corrected phenotypes of the validation group.

## 3. Result

### 3.1. Estimates of Variance Components and Heritability

For the five traits, we decomposed the total genetic variance into the additive and dominance variances. The corresponding components are shown in Table 2. Based on the additive and dominance model, for CW, DP, MP, ADG, and CR, the broad-sense heritabilities ($h_{a}^{2}+h_{d}^{2}$) were 0.578, 0.391, 0.318, 0.298, and 0.314, and the narrow-sense heritabilities were 0.42, 0.23, 0.267, 0.256, and 0.217, respectively. Based on the additive model, the heritabilities were 0.439, 0.252, 0.244, 0.294, and 0.248, respectively.

### 3.2. Goodness of Fit

Goodness of fit estimations based on likelihood ratio tests are shown in Table 3. GBLUP-D had a larger log likelihood than GBLUP, which means that GBLUP-D fit the data better. On the other hand, GBLUP-D was significantly (*p* < 0.05) superior to GBLUP. The results indicated that the goodness of fit was improved by including dominance effects in the model.

### 3.3. Predictive Ability

The predictive abilities of the estimated genomic breeding values for five traits using the five-fold cross-validation method with BayesA, BayesAD, GBLUP, and GBLUP-D are shown in Table 4. The predictive ability of these five traits ranged from 0.239 to 0.431 and from 0.244 to 0.438 for GBLUP and GBLUP-D, respectively. For BayesA and BayesAD, the predictive abilities ranged from 0.244 to 0.403 and from 0.249 to 0.411, respectively. We found that the predictive ability was slightly better for models including dominance effects compared to additive-only models. For instance, the predictive ability of BayesAD increased by 1.1%, 0.8%, 0.5%, 0.8%, and 0.6% compared with BayesA for CW, MP, CR, DG, and DP, respectively. Similarly, the predictive ability of GBLUP-D increased by 0.9%, 0.8%, 0.5%, 0.7%, and 0.5%, respectively, compared with GBLUP. Moreover, the predictive ability of BayesAD was higher than that of GBLUP-D for most of the traits.

### 3.4. Genome-Wide Association Studies of Additive and Dominance Effects

**Figure 1:**
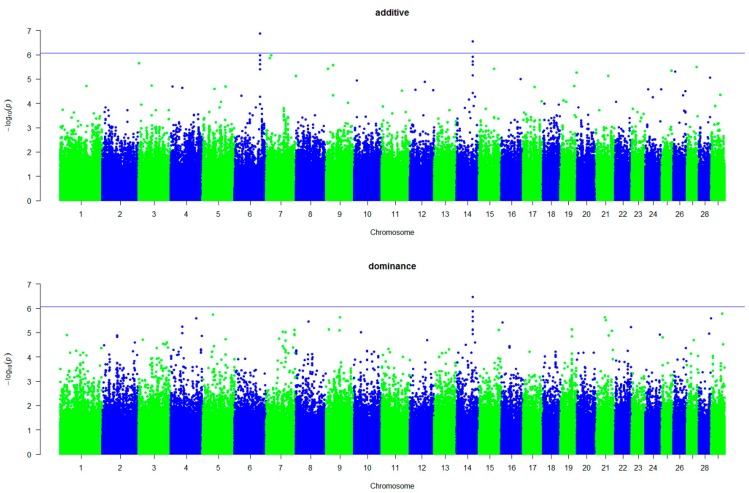
Manhattan plots showing the significant single nucleotide polymorphisms (SNPs) associated with carcass weight with additive and dominance effects. The X-axis represents chromosomes and the Y-axis indicates −log^10^ (*p*-value).

**Figure 2:**
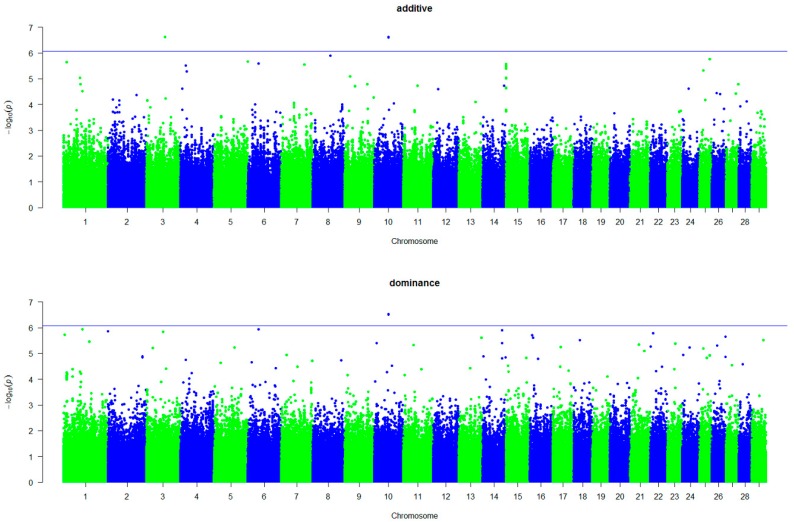
Manhattan plots showing the significant single nucleotide polymorphisms (SNPs) associated with average daily gain with additive and dominance effects. The X-axis represents chromosomes and the Y-axis indicates −log^10^ (*p*-value).

We performed a whole-genome single-marker scan for additive and dominance effects with five traits in 1233 Simmental using a multiple-locus mixed-model. We found 15 significant additive signals and 10 significant dominance signals (*p* < $8.37\times 10^{-7}$) associated with the five traits (Table 5). Manhattan plots for CW with additive and dominance effects are shown in Figure 1. We observed that one of the top SNPs, BovineHD1400017459, showed a strong additive and dominance association with CW. This SNP was embedded in regulating synaptic membrane exocytosis 2 (*RIMS2*) in BTA14. We also observed that two top SNPs, BovineHD1000015632 and BovineHD1000015492, both showed a strong additive and dominance association with ADG (Figure 2), and these two SNPs were embedded in aldehyde dehydrogenase 1 family member A2 (*ALDH1A2*). Manhattan plots of the results for DP, MP, and CR with additive and dominance effects were shown in Supplementary Materials (Figures S1–S3). We identified six significant SNPs associated with DP, which were distributed on six chromosomes. These SNPs were within or close to G protein-coupled receptor 68 (*GPR68*), guanylate cyclase 1 soluble subunit alpha 2 (*GUCY1A2*), abl interactor 1 (*ABI1*), armadillo repeat containing 1 (*ARMC1*), SKI family transcriptional corepressor 2 (*SKOR2*), and STAM binding protein like 1 (*STAMBPL1*). Four of these SNPs contributed to both additive and dominance effects. One SNP was associated with MP near *RIMS2*. However, we did not detect any significant dominance signal for MP. We observed three SNPs, within or near three genes, that were associated with CR. Interestingly, we exclusively detected the other four SNPs when considering the dominance effects.

## 4. Discussion

This study provided a systematic view of dominance effects through a comprehensive analysis of CW, DP, MP, ADG, and CR in Simmental beef cattle, including an analysis of variance components, genome-wide association studies and genomic prediction. For these traits, the estimates of dominance variance illustrated that dominance genetic effects had variable degrees of contribution to the total genetic variance. Moreover, we confirmed that BayesAD was superior to GBLUP-D in genomic prediction for most of the studied traits, when the model included additive and dominance genetic effects. We found 15 significant additive signals and 10 significant dominance signals for the five traits in the genome-wide association studies. Among these significant signals, six SNPs contributed to both additive and dominance effects. 

### 4.1. Additive and Dominance Genetic Variance

Genome-wide dense markers can provide a feasible method to detect non-additive genetic variance and predict the merit of complex traits. Additive genomic relationship matrix [20,21] has been widely applied for genomic prediction with linear models [22,23,24]. The current study firstly illustrated the method to construct a dominance relationship matrix using SNP markers in Simmental beef cattle. Compared to the pedigree-based relationship matrix, the genomic relationship matrix can capture both the Mendelian segregation and genetic links through unknown common ancestors [1]. Moreover, the genomic relationship matrices are applicable for different populations with or without pedigree information, which is particularly advantageous for population genetics studies on farm animals [25].

Across traits, the proportion of phenotypic variance due to dominance values varied widely. The estimates of the dominance variance as a proportion of phenotypic variance were 4.2%, 5.1%, 9.7%, 15.8%, and 16.1% for ADG, MP, CR, CW, and DP, respectively. No studies have been reported to decompose dominance variation components of these traits in the Simmental population. There are a few publications that have reported estimates of the dominance genetic variance for some traits related to our study in other population. For example, Su et al. [1] estimated dominance genetic variance for average daily gain in pigs using high-density SNPs, and reported that 5.6% of total phenotypic variance was explained by dominance variance, which is higher than our estimate of 4.2%. In nine different beef cattle populations, the estimates of dominance variance as a proportion of phenotypic variance was 18% for carcass retail beef yield [5], which is similar to our result. These results indicated that dominance genetic variations are important in genetic variance components estimation, and are different for various traits and populations.

### 4.2. Genomic Prediction of Complex Traits

The current study showed that dominance genetic variance estimation was feasible for complex traits. Therefore, it was expected that a model including dominance genetic effects could increase the predictive ability. In this study, predictive abilities of genomic estimated breeding values using models including both additive and dominance effects were improved by 0.5–1.1% compared to those only using additive genetic models. Compared to the large dominance genetic variance and the detection of dominance signals, the gain in predictive ability of genomic selection by including dominance effects in the prediction model was relatively small. We identified two significant dominance SNPs and found that the proportion of dominance variance to phenotypic variance was 0.158 for CW. The accuracy of genomic estimated breeding value was increased by 0.011. However, for MP, we did not detect any significant dominance signals and the ratio of dominance variance to phenotypic variance was merely 0.051, while the predictive ability was only improved by 0.05. For DP and CR, we witnessed moderate accuracy improvements (0.008 and 0.006) with approximately the same number of significant dominance SNPs and ratio of dominance variance to phenotypic variance. However, for ADG, the accuracy improvement was relatively high with a low dominance variance ratio. These observations may be attributable to a few factors: (1) incomplete decomposition of dominance variance from total genetic variance, and (2) lack of full-sib pairs between reference and prediction populations because full-sibs are the primary source of non-additive relationships [7].

In Danish Duroc pigs, the accuracies of predicted breeding values of average daily gain were 28.5% and 29.2% for a model without dominance effects and a model with dominance effects, respectively, and the results showed that the accuracy increased by only 0.7% by using GBLUP-D [1]. In Holstein and Jersey cattle, compared with a model only including additive effects, the predictive ability for yield traits increased by 0.6%–1.1% using GBLUP-D [4], and the increased accuracies were consistent with the findings of our study. In a nucleus pig line, the predictive abilities increased by only 0.2–0.7% using GBLUP-D compared to GBLUP [6]. The inclusion of dominance effects increased the accuracy of genomic breeding values by 2% in the offspring by using BayesAD in a simulation dataset [16]. By using fast BayesB method including dominance effects in a simulation study, prediction accuracy could be improved by 1.3–2% [26]. However, in a Fleckvieh cattle population, the predictive ability of breeding value was not improved when dominance effects were included in GBLUP-D [27]. Similarly, for nine different populations of three cattle breeds in Australia, no significant improvement from including dominance deviations was observed for any traits by using GBLUP-D [5]. Large variations have been observed in terms of the predictive ability across different studies, which may reflect the different features of various traits across populations. We applied GBLUP-D and BayesAD to estimate genetic parameters and compare prediction accuracies. We obtained better results using BayesAD compared to GBLUP-D for most of the traits. GBLUP-D assumed that the effects of all SNPs are normally distributed with equal variance. This model may not be satisfactory in the situation where few markers have null effect or a very small effect [1]. However, BayesAD assumes additive and dominance effects following a heavier tailed Student’s t-distribution. Since the Student’s t-distribution approximates the normal distribution when the degree of freedom (*v*) increases, GBLUP can be considered as a limiting case of BayesA [16]. Although the improvement of prediction is not relatively high when the model including dominance effects, we cannot ignore the potential application to animal breeding programs. For example, the BayesAD model could allow mating allocation to exploit dominance. Additional data can be obtained if mating allocation techniques are implemented using an appropriate design for future matings [28].

### 4.3. GWAS with Additive and Dominance Effects

We performed GWAS in 1233 Simmental beef cattle to systematically detect additive and dominance variants for CW, DP, MP, ADG, and CR, and we identified 15 additive and 10 dominance signals within or near 13 and 9 genes, respectively. We observed one significant additive signal extragenic SNP near fibroblast growth factor 5 (*FGF5*) on BTA6 and one intragenic SNP (BovineHD1400017459) located in regulating synaptic membrane exocytosis 2 (*RIMS2*) on BTA14, which were associated with CW. *FGF5* had relationships with embryonic development, cell growth, morphogenesis, tissue repair, tumor growth, and invasion [29], and it may be a potential candidate gene associated with CW. Several studies have reported that *RIMS2* is associated with carcass weight in Simmental beef cattle [30,31,32]. This gene was also reported as a candidate gene associated with Paget’s disease of bone [33]. We also identified six additional loci near six genes located on BTA13, BTA14, BTA15, BTA21, BTA24, and BTA26, respectively, which were significantly associated with DP. These six genes were mostly reported to have associations with growth traits. For example, *GPR68* has been confirmed as a candidate gene for growth traits of Charolais beef cattle [34]. *GUCY1A2* plays a critical role in human blood pressure [35,36]. *ARMC1* was reported as a candidate gene associated with body weight, body length, body height, and chest circumference in Chinese Laiwu pigs [33]. It has been reported that this gene is related to obesity-related traits in humans [37]. *SKOR2* has been reported as a candidate gene for changes in weight and BMI in male and female adolescents [38]. *STAMBPL1* was reported as a candidate gene for lung vasculature and immune cell functions in 26 inbred mouse strains [39]. We observed that one SNP (BovineHD1400017459) located in *RIMS2* on BTA14 was associated with MP. Three SNPs located in two genes were identified as being associated with ADG; one of these genes was ST6 N-acetylgalactosaminide alpha-2,6-sialyltransferase 5 (*ST6GALNAC5*) on BTA3, while the other gene was aldehyde dehydrogenase 1 family member A2 (*ALDH1A2*) on BTA10. It was reported that *ST6GALNAC5* is closely related to fat accumulation in human and pigs [40]. *ALDH1A2* has been identified to play critical roles in the synthesis of retinoic acid, the active derivative of vitamin A (retinol), which mainly affects body growth and bone development in mice [41]. Three SNPs near to or embedded in three genes were identified as being associated with CR. These three genes were mercaptopyruvate sulfurtransferase (*MPST*) on BTA5, member RAS oncogene family (*RAB3B*) on BTA3, and neuron navigator 3 (*NAV3*) on BTA5, respectively. *MPST* was a protein coding gene, and it had been reported as a candidate gene associated with protein production traits in dairy cattle [42]. Some of genes like *ABI1*, *RAB3B*, and *NAV3* have not been reported associated with our studied traits in beef cattle. However, these genes may play important roles in some metabolic pathways related to DP and CR. 

We observed that six significant dominance signals were both characterized as additive and dominant for CW (one SNP), DP (three SNPs), and ADG (two SNPs). The other four significant dominance signals were only associated with CR. Three SNPs were respectively located in ryanodine receptor 3 (*RYR3*) on BTA10, echinoderm microtubule-associated protein like 6 (*EML6*) on BTA11, and homer scaffold protein 1 (*HOMER1*) on BTA10, and one SNP was located in upstream of sterile alpha motif domain containing 12 (*SAMD12*) on BTA14. This may be explained by that these four signals only contribute to dominance variance. The mode of gene action of these SNPs seems purely dominant as the dominance variance in proportion to additive genetic variance of CR was as large as 44%, which indicated that dominance plays an important role in the genetic architecture. However, the effect of these significant signals needs to be further confirmed based on independent studies. Interestingly, we found no significant SNPs in dominance effects association studies for meat percentage. Due to a possible sparseness of dominance effects, MLMM may under-perform for association mapping. Future research is needed to develop more association models for dominance effects.

## 5. Conclusions

We comprehensively evaluated the contribution of dominance effects to five important traits related to growth and carcass in Chinese Simmental beef cattle through analysis of the proportion of dominance variance to the phenotypic variance, genome-wide association studies, and genomic prediction. We found that the proportion of dominance variance to phenotypic variance was 4.2%–16.1% for these five traits, and the inclusion of dominance genetic effects in genomic prediction models can slightly improve the accuracy of genomic breeding values. We also observed that 15 additive and 10 dominance signals were significantly associated with these traits. Our findings could be helpful for further studies of causal mutation mapping and genomic selection models with dominance effects in Chinese Simmental beef cattle. 

## Figures and Tables

**Table 1:** Summary statistics of phenotypes for the five traits in the Simmental population.

Trait	N	Mean	SD	Max	Min
CW (kg)	1233	271.87	45.41	486	162.62
DP	1233	0.54	0.028	0.68	0.41
MP	1233	0.45	0.31	0.61	0.32
ADG (kg)	1233	0.97	0.22	2.41	0.38
CR (kg)	1233	11.59	3.33	28.68	4.5

CW: carcass weight; DP: dressing percentage; MP: meat percentage; ADG: average daily gain; and CR: chuck roll.

**Table 2:** Variance components of genotypic additive and dominance values for the five traits.

Heritability/Variance	CW	DP	MP	ADG	CR
$\sigma_{a}^{2}{}^{AD}$	415.642	0.000133	0.000181	0.00147	1.077
$\sigma_{d}^{2}{}^{AD}$	156.579	0.0000932	0.0000343	0.000242	0.482
$\sigma_{e}^{2}{}^{AD}$	417.453	0.000352	0.000462	0.00402	3.394
$h_{A}^{2}{}^{AD}$	0.420	0.230	0.267	0.256	0.217
$h_{D}^{2^{AD}}$	0.158	0.161	0.051	0.042	0.097
${\frac{\sigma_{D}^{2}}{\left(\sigma_{D}^{2}+\sigma_{A}^{2}\right)}}^{AD}$	0.273	0.412	0.159	0.141	0.309
$\sigma_{a}^{2}{}^{A}$	437.036	0.000142	0.000169	0.00165	1.236
$\sigma_{e}^{2}{}^{A}$	557.988	0.000421	0.000523	0.00397	3.741
$h_{A}^{2}{}^{A}$	0.439	0.252	0.244	0.294	0.248

AD: model based on additive and dominance effects of markers; A: model including additive effects only; CW: carcass weight; MP: meat percentage; CR: chuck roll; ADG: average daily gain; and DP: dressing percentage.

**Table 3:** Estimates of log likelihood, *χ*^2^ value, and the corresponding *p*-value of likelihood ratio.

Likelihood Statistic	Model	CW	DP	MP	ADG	CR
Log(L)	GBLUP	−3799.82	3874.55	3810.34	3873.79	−1473.89
	GBLUP-D	−3799.68	3874.67	3810.47	3873.93	−1473.75
$\chi^{2}$-value ^a^	GBLUP-D	3.91	3.54	3.86	3.87	3.68
*p*-value		0.03	0.05	0.04	0.04	0.05

^a^$\chi^{2}$ = −2ln (likelihood for genomic best linear unbiased prediction (GBLUP)/likelihood for GBLUP-D); CW: carcass weight; DP: dressing percentage; MP: meat percentage; ADG: average daily gain; and CR: chuck roll.

**Table 4:** Predictive abilities of the five traits using different models including additive and dominance effects.

	CW	DP	MP	ADG	CR
BayesA	0.262	0.262	0.244	0.403	0.375
BayesAD	0.273	0.270	0.249	0.411	0.381
GBLUP	0.253	0.251	0.239	0.431	0.369
GBLUP-D	0.262	0.259	0.244	0.438	0.374

CW: carcass weight; DP: dressing percentage; MP: meat percentage; ADG: average daily gain; and CR: chuck roll.

**Table 5:** Significant additive and dominance SNPs identified for five traits.

Trait	SNP	A/D	BTA	Position ^a^	Distance ^b^	Gene Name ^c^	*p*-Value ^pa^	*p*-Value ^pd^
CW	BovineHD0600026881	A	6	96,746,323	−183	*FGF5*	1.33 × 10^−7^	-
CW	BovineHD1400017459	A + D	14	62,788,724	within	*RIMS2*	2.83 × 10^−7^	3.41 × 10^−7^
DP	BovineHD2100016298	A + D	21	56,606,414	15,215	*GPR68*	2.41 × 10^−7^	4.08 × 10^−7^
DP	BovineHD1500004272	A + D	15	16,715,193	within	*GUCY1A2*	1.53 × 10^−7^	3.83 × 10^−7^
DP	BovineHD1300005158	A	13	18,099,016	within	*ABI1*	4.67 × 10^−7^	-
DP	BovineHD1400009103	A + D	14	31,569,541	128,114	*ARMC1*	4.96 × 10^−7^	4.01 × 10^−7^
DP	BovineHD2400013194	A	24	47,440,919	−43,561	*SKOR2*	5.93 × 10^−7^	-
DP	BovineHD2600002715	A	26	10,622,551	5333	*STAMBPL1*	6.84 × 10^−8^	-
MP	BovineHD1400017459	A	14	62,788,724	within	*RIMS2*	5.26 × 10^−7^	-
ADG	BovineHD0300020097	A	3	3,948,572	within	*ST6GALNAC5*	2.41 × 10^−7^	-
ADG	BovineHD1000015632	A + D	10	52,349,382	within	*ALDH1A2*	2.35 × 10^−7^	2.91 × 10^−7^
ADG	BovineHD1000015492	A + D	10	52,349,964	within	*ALDH1A2*	2.53 × 10^−7^	3.07 × 10^−7^
CR	BovineHD0500036083	A	5	75,823,709	within	*MPST*	4.98 × 10^−7^	-
CR	BovineHD0300027330	A	3	94,958,653	within	*RAB3B*	3.54 × 10^−7^	-
CR	BTB-00216812	A	5	7,314,405	79,333	*NAV3*	2.23 × 10^−7^	-
CR	BovineHD1000009483	D	10	28,879,512	within	*RYR3*	-	1.51 × 10^−7^
CR	BovineHD1100011059	D	11	37,361,510	within	*EML6*	-	1.91 × 10^−7^
CR	BovineHD1000003443	D	10	10,362,838	within	*HOMER1*	-	3.42 × 10^−7^
CR	BovineHD1400013490	D	4	47,683,093	96,642	*SAMD12*	-	2.13 × 10^−7^

SNP: Single-nucleotide polymorphism name in the Bovine HD panel; A/D: A for additive effects, D for dominance effects; ^a^: position (bp) on UMD3.1; ^b^: the location of SNP in upstream/downstream of the nearest gene and within means that the identified SNP is embedded in gene; ^c^: name of the nearest gene; ^pa^: *p*-values calculated from the multi-locus mixed-model analysis for additive effects; ^pd^: *p*-values calculated from the multi-locus mixed-model analysis for dominance effects; CW: carcass weight; DP: dressing percentage; MP: meat percentage; ADG: average daily gain; and CR: chuck roll.

## Supplementary Materials

The following are available online at https://www.mdpi.com/2076-2615/9/12/1055/s1, Figure S1: Manhattan plots showing the significant single nucleotide polymorphisms (SNPs) associated with chuck roll with additive and dominance effects. The X-axis represents chromosomes and the Y-axis indicates −log_10_ ($8.37\times 10^{-7}$). Figure S2. Manhattan plots showing the significant single nucleotide polymorphisms (SNPs) associated with dressing percentage with additive and dominance effects. The X-axis represents chromosomes and the Y-axis indicates −log_10_ ($8.37\times 10^{-7}$). Figure S3. Manhattan plots showing the significant single nucleotide polymorphisms (SNPs) associated with meat percentage with additive and dominance effects. The X-axis represents chromosomes and the Y-axis indicates −log_10_ ($8.37\times 10^{-7}$).
