# Supplementary material for: Genomic Prediction and Association Analysis with Models Including Dominance Effects for Important Traits in Chinese Simmental Beef Cattle
Source: Animals (Basel). 2019 Dec 1;9(12):1055. doi: 10.3390/ani9121055 (PMC6941016; doi:10.3390/ani9121055)
Supplement: Supplementary file 1 [file animals-09-01055-s001.pdf]

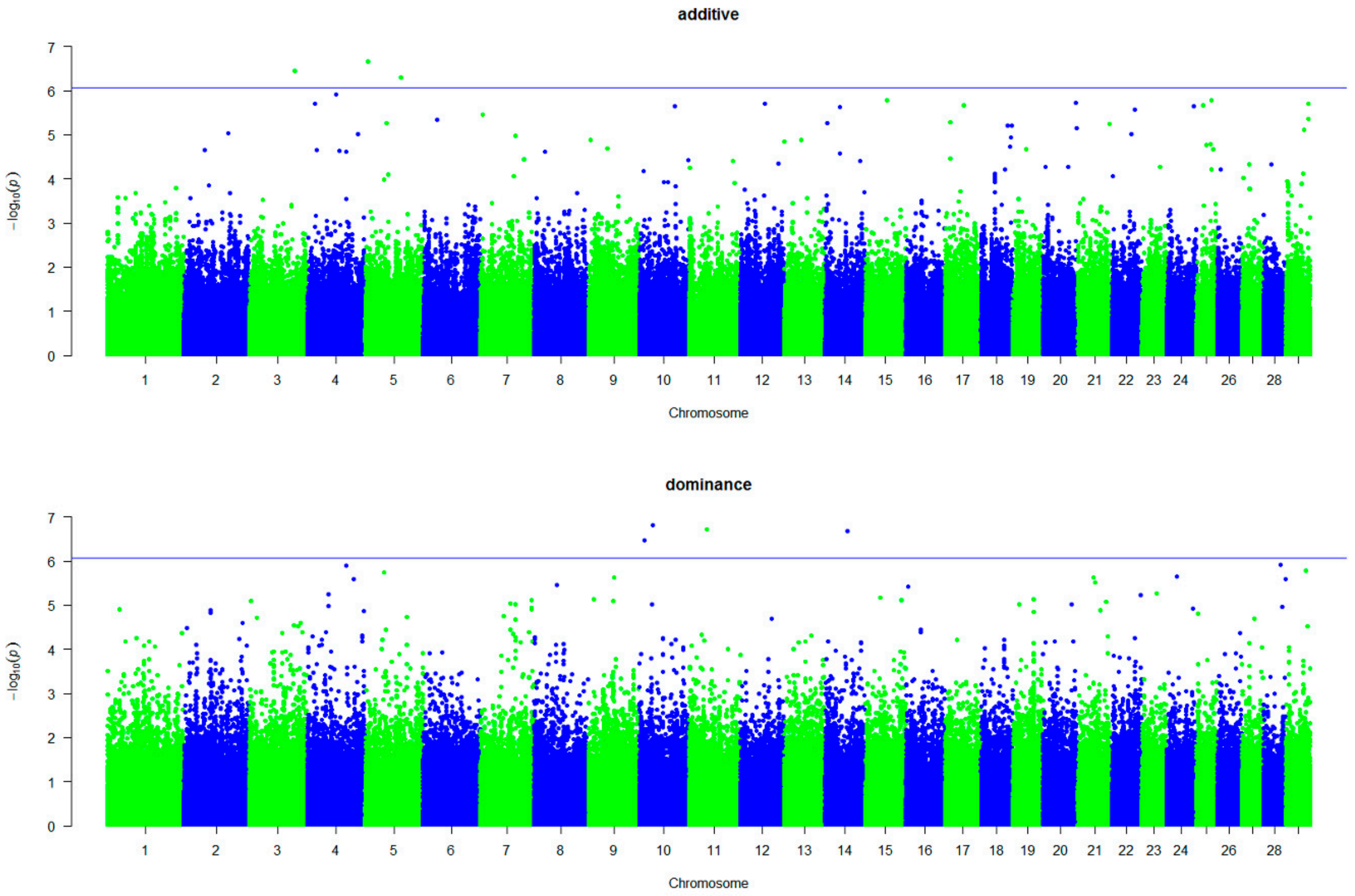

Figure S1. Manhattan plots showing the significant single nucleotide polymorphisms (SNPs) associated with chuck roll with additive and dominance effects. The X-axis represents chromosomes and the Y-axis indicates $-\log_{10}$ (P-value).

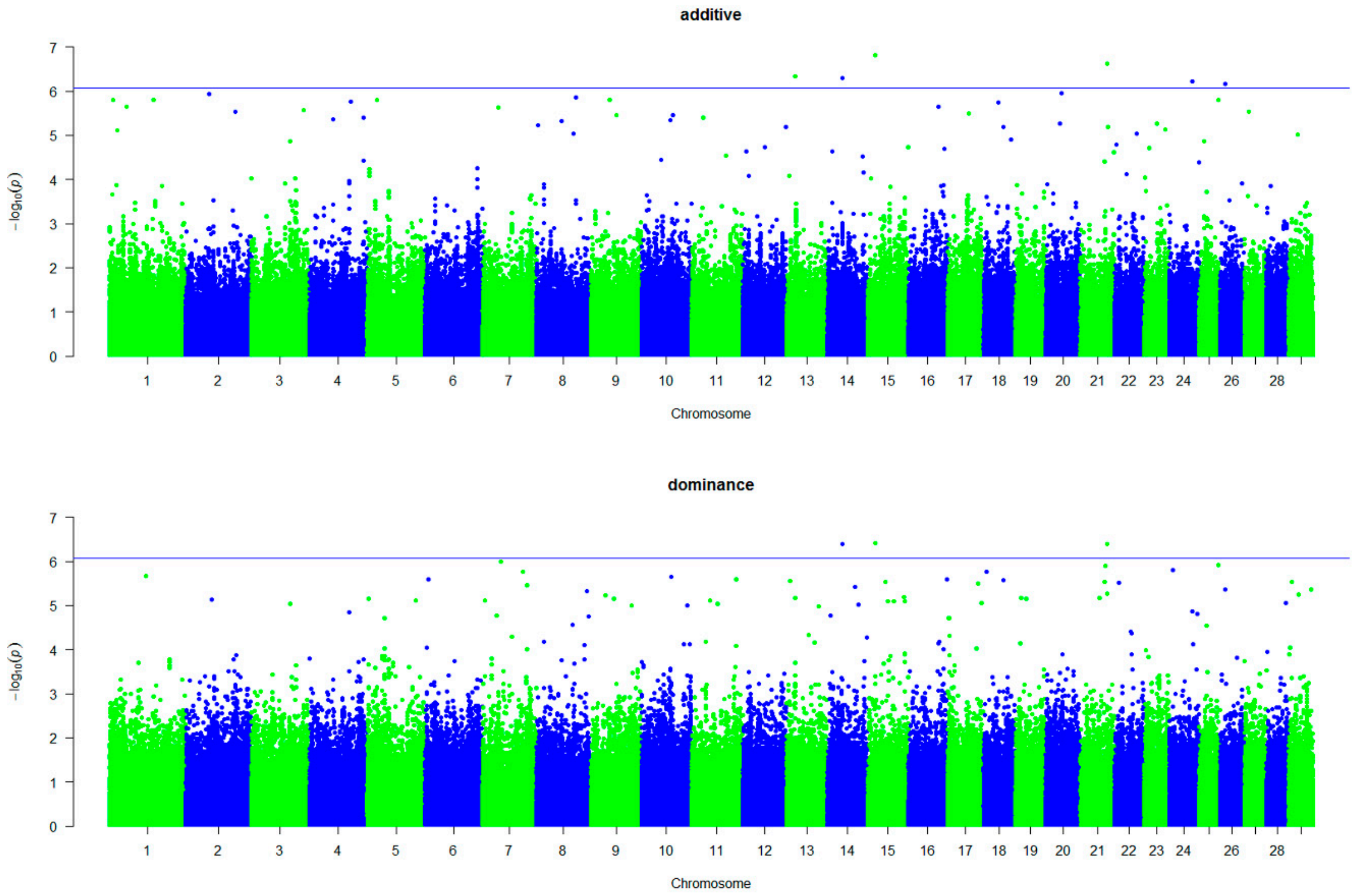

Additional Figure S2. Manhattan plots showing the significant single nucleotide polymorphisms (SNPs) associated with dressing percentage with additive and dominance effects. The X-axis represents chromosomes and the Y-axis indicates $-\log^{10}$ (*P*-value).

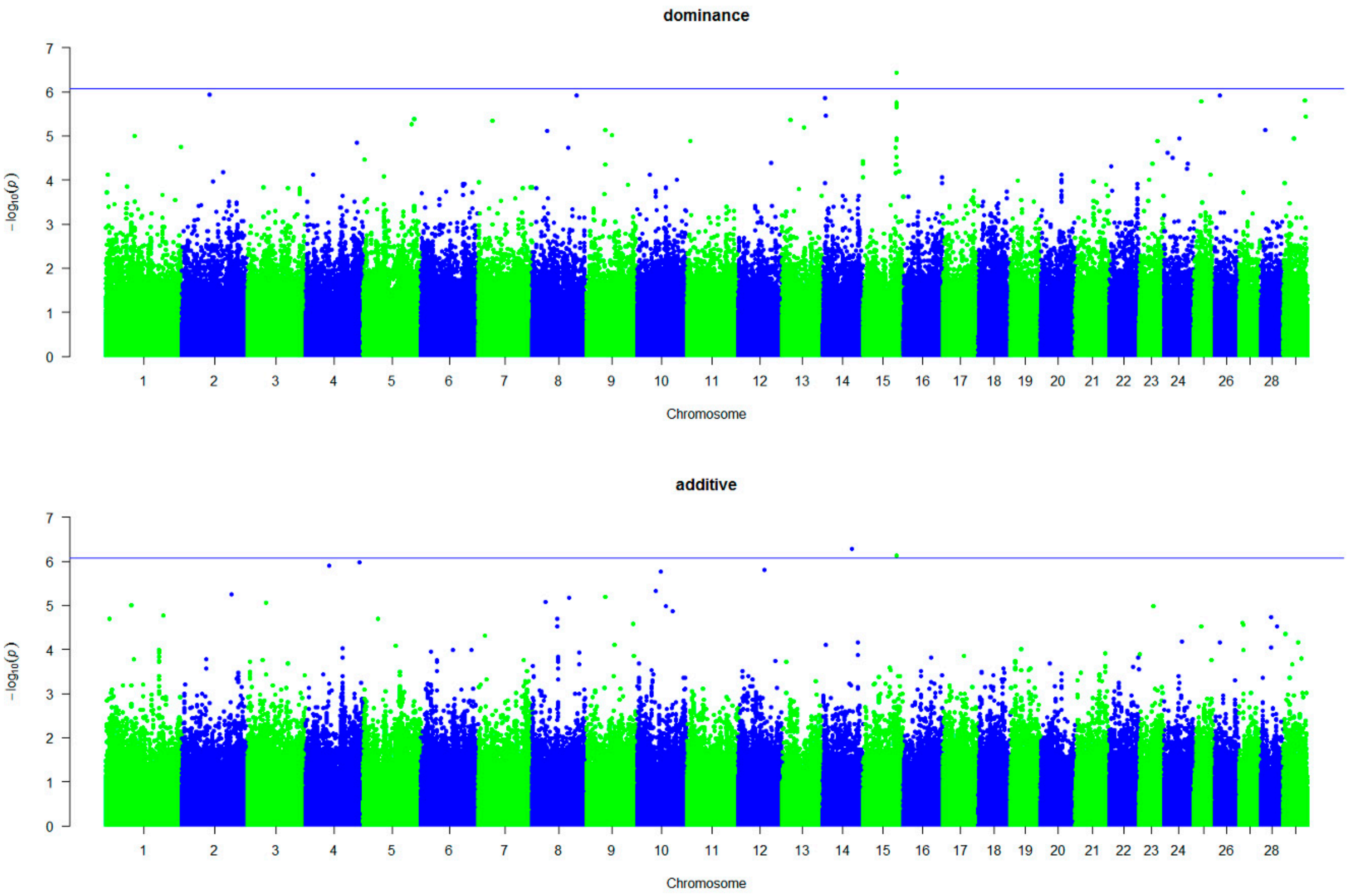

Additional Figure S3. Manhattan plots showing the significant single nucleotide polymorphisms (SNPs) associated with meat percentage with additive and dominance effects. The X-axis represents chromosomes and the Y-axis indicates $-\log^{10}$ (*P*-value).
